# Address in Surgery, Delivered at the Annual Meeting of the British Medical Association

**Published:** 1865-01

**Authors:** J. Paget


### CHRONICLE OF MEDICAL SCIENCE.

*Address in Surgery.* Delivered at the Annual Meeting of the British Medical Association. By J. PAGET, F. R. S.

*Mr. President and Gentlemen*:—I have chosen as the subject of my address, "The Treatment of Patients after Surgical Operations," and I venture to think that I shall best discharge my duty if I deal with this subject, not merely in retrospect, as if there were some great achievements over which we might rejoice, but rather with the view of indicating some of the things which yet remain to be done, and which may be done if the members of this great Association will make it their chief business to do them. There is, indeed, nothing in the retrospect from which we need avert our faces; rather, there are many things over which we might boast as good work achieved; and if I had to name them all, or illustrate the chief of them by one, I would take the security and soundness of our practice, founded on a wider recognition of the principle that the recovery from an operation, as from any other injury, is so natural a process, that, except in certain exceptional cases, it should not be at any time or in any way interfered with; for we are so constructed that injuries by violence, from whatever source, do of themselves, and naturally, bring about the processes for their own amendment. We are fitted, not only for the calm of life, but for the storm of it; not only for the things which are certain, but for those which are probable: nay, almost for all that are possible as events that occur in our life; and among these probable events are injuries by violence. Among these, and not widely different from them, except in regard to some of the conditions in which patients come before us, are the injuries inflicted by surgical operations.

Nevertheless, whatever be our faith in the natural process of recovery from injuries, there are certain things left for us to do or to watch, or in some respects to guide. And first of all we have to decide in every case the method by which the surgical injury shall be healed. We have long been settled in a just preference for the most speedy mode of union, that is, for union by the first intention, whether there be healing by immediate union or by the union by adhesion. And there are many obvious reasons for this; but the most potent of them, to my mind, is, that so long as a wound is unhealed, there is some risk, however small, that the patient may fall into pyæmia or erysipelas, or some other of the sore plagues of surgery. But the manner of healing the wound being decided, there yet remain many things to be done, even by those who have the most full faith in the natural processes of recovery. They may, however, perhaps be summed up in comparatively very few words—namely, repose and cleanliness. But both of these leave much still to be done. For repose there must be not only perfect quietude of the wounded part and the parts of the wound itself, but of all the circumstances that surround the patient; and for cleanliness the most complete purity of the air, the water, and everything that may come near the wound.

And repose requires much more than this. It requires the absolute non-interference, except on express occasions, of the surgeon himself. There must be no rough contacts, no searchings, no pressure, no touchings of the wound except with the softest things, and in the softest ways, such as soft streams of water. More than this will spoil many a good day of nature's work. And for cleanliness, there must be not only those things which are commonly provided, but much more—things on a larger scale—baths, either general or local baths, the frequent changings of dressings, if there are to be any dressings at all, and the frequent changes, not only of bed linen, but of beds, and even in hospitals, the frequent changes of wards, or places in wards, or, in private houses, frequent changes of the rooms that the patient must at each time occupy; and in all these things the surgeon has to set before himself to be done, a great many things that do not lie commonly enumerated amongst the general rules for surgery. And simple as the rules may seem, they include yet many things, but I can speak of them only in general terms. Nevertheless, the very generality of the terms may imply a largeness of the rule that may be laid down, that for all the ordinary management of patients after operations, repose and cleanliness are the only two things that need to be observed, and this not only for the local management of the wound, but even for those things which accompany it, namely, the shock and the re-action, that are either associated with or follow after other more important operations; for deaths from mere shock are certainly very rare, and the results of re-action are, I believe, never fatal, even when it runs up into the condition of acute traumatic fever. The discomfort of a sharp re-action after a shock is perhaps too apt to make us look upon it as a kind of disease; and yet, if we would judge it more truly, it is really only an example of the higher and more peculiar power of organic bodies. In inorganic matter the axiom is that action and re-action are opposite and equal. In organic bodies it is not so: the re-action is always, in health, stronger than the action.

The heart, for example, if it be depressed in its action, suppose by the shock of a sudden shower of water, recovers, not the condition which it had before, but something beyond it. The spring recoils with the same force as that which depressed it; the pendulum swings equal distances this way and that; but the heart with vital force recovers to something more than the force which depressed it. So a muscle which is gradually wasted by exercise recovers, not only from the waste, but to something more of power, and becomes hypertrophied. In a similar manner, after a shock, all the parts which have been depressed in their vital functions, suspended, or brought to their lowest mark, recover not merely to the normal standard in which they worked before, but to something beyond that standard; they re-act with more force than they had even before they were depressed. I repeat, therefore, that however much the phenomena of re-action, even in sharpest form, may simulate disease, they must be regarded and dealt with as those which are the most manifest indications of perfect and strong health; hypertrophies, as we call them—diseases that indicate only the perfection of the vital power in that, being depressed, it does not recover like an inorganic spring or pendulum, but rises to something more than the force which is exercised at some previous time. In this way, however much we may regard the forces of the natural process of recovery, and that they leave us really with comparatively very little to do in the ordinary progress of our surgical cases, yet there is enough left for us to be done in the cases that pass beyond these; for in regard even to the management of the general phenomena, questions continually arise as to what must be done to meet this or that condition. I think the distinction may be drawn safely according to the question of whether they be events that are natural to the body, and not avertable, or such as come to the body from without, or are bred in it by error from within, and which need, therefore, our most careful search. For with regard to the questions of general management, the single fact of cleanliness, if we take it in its largest sense, will shut out nearly all the mischiefs that come from without; but that cleanliness must be cleanliness of person, of air, of water, and of everything that comes near. And so with diet: On this there may be more question, at least there has been more question ; and I venture to think that if all are not agreed, yet all will be soon completely agreed, for the pendulum of general opinion which swung to the other extreme some thirty years ago, and very nearly swung to the opposite extreme some three or four years ago, is now setting back to the more just medium of moderation. I remember that one of the first cases I ever took, some thirty years ago, was that of a man who had the greater part of the lower jaw removed for a fibrous tumour. It was long before the days of chloroform. The cutting part of the operation occupied upwards of an hour; the patient remained on the table more than an hour and a half, and he left at the close of the operation greatly exhausted; and though he was a young and very healthy man, for the three days following the operation he received three table-spoonfuls of milk per diem, and about a pint of milk-and-water was on each day injected into the rectum. The rest of the diet for the remainder of the case was after the same scale. He recovered with remarkable quickness, and without one hindrance to his good progress; and I remember that the operator, in a clinical lecture upon the subject, said he ascribed so remarkable a recovery to the great abstinence that was used. The diet in such cases, he said, could not be too low. At the present time, perhaps, in some places, certainly in many places not long ago, the same patient would have been plied with wine, brandy, and with all manner of food ; and I have little doubt that he would have recovered as well, as speedily, and as perfectly, for I have seen the same oppositions of practice carried out without mischief in the last few years.

But then, what is the reason of this apparently equal success of two opposite methods of treatment? Certainly not that they are adapted to different times or different types of disease ; for I repeat that I have within my own experience the last few years seen or practised them, both without damage ; but that between the two there runs the natural process of recovery, which is, in health, in all persons not already tainted with great disease, so certain, that neither excess nor deficiency of diet, nor surfeit nor starvation, can always avert it. Let me not seem to be indifferent as to how a patient should be fed after surgical operation. I cite these apparently equal successes of different modes of treatment, not for the praise of either. I have no doubt that either of them, always adopted, would sometimes do great mischief; but taken together, and with the evidence afforded by cases that are left alone, they are very strong tests and very strong proofs of the sufficiency of the natural process of recovery; that if they be left only with such rules of prudence, of personal cleanliness, of sufficient, even of liberal, mixed diet, it is, for all the ordinary cases that will come within our charge, amply sufficient not only for the repair of the wound, but for the recovery from those general injuries which patients sustain by operation.

I think, therefore, we may boast—it may be we have to regret that the boast could not come sooner—we have to boast that we can now treat our patients, after operations, in a natural and reasonable way, in full reliance on that which is one of the first principles of our construction—that we are made to bear, not only the stress of all the ordinary events of life, but the stress of injuries; prepared, as I said before, not only for the calm, but for the storms of life. And herein I think that surgery has made a considerable contribution to that study of the natural history of disease which is becoming one of the most pressing wants of our time. What will happen if this and that disease be left to itself, or only just so managed as the comfort of the patient, or his obvious needs may suggest ? The question has been asked very often, but it is very rarely answered ; and yet it must be answered before we can judge rightly of the value of any medical or surgical treatment. Till we have made our standards of what the progress of disease is if left alone, we cannot judge of our own power in controlling, or amending, or remedying it. Surgery has done this in regard to injuries. And let it not be thought that, by thus reducing the practice of surgery, in ordinary cases of operations, to the mere watching and guarding of the mischiefs that may happen, we thereby diminish the value of the profession, or degrade it. The obstetrician has not done so by giving up the meddlesome midwifery of former ages, and, certainly, that branch of our profession was never more honored nor more useful than it is now, when it confines itself in all ordinary cases to the watching of the perfect ways of nature.

So let the surgeon stand by and watch ; let him him be content if he can keep out mischief—always a very difficult task. Let him be well content if he can do that which is more difficult—control the restlessness of popular or half-informed ignorance, which would be perpetually, either in its audacity or its fright, endeavoring to amend the perfect ways of nature. Or if he be ambitious of more than this, he need not wait long, for easy as our course may seem when all runs smooth, we soon find that the whole grasp of our knowledge and the whole strain of our mental power is not more than enough to meet the difficulties of the case, when anything runs counter to the common course of nature.—Now let me speak of some of these, and chiefly with the view of indicating what may be the chief subjects and the chief course of our future study of them. I mean, namely, those cases which do not proceed thus smoothly and quietly according to the common course of things, in which every patient submitted to an operation, should recover, as our common expression is, without one bad symptom. And here I must say generally, that it seems to me that what we chiefly need is a more minute and exact record of all our cases—of cases, namely, not only when the operation ends in death, but of those in which it brings in any mischief—even some of the smallest.

There has been excellent work, and most laborious done, in tabulating the cases of the mortalities after operations, and it has been done so well that it seems ungracious to say that it is time now we should be doing something more or something better. And yet it is certain that we do want to know how, not merely the total mortalities of any considerable operation, but in each hospital, in each private practice, in each place, and after each mode of treatment, not only the whole amount of mortality, but the particular causes which have led to it; nor only so—the particular causes, if we can ascertain them, which have led to hindrance in the ordinary process of recovery. Now, first there are certain cases which confuse our records in that they ought never to be mixed up with the results of operations as being, in any sense, due to them—namely, cases of hernia, tracheotomy and trephining; for of all these we may say the operation itself does not cause death; the only fault to be ascribed to it is, that it does not always save life.

But we have no right to reckon these as cases due to operations. Every surgeon who has performed these operations has had to regret a very large mortality from them; but mortality has not been because of anything that he has done, but because solely of the insufficiency of his means. For myself, among all the cases of this description that I have operated upon, I am not sure that I have ever lost a patient directly and truly as a consequence of the operation; yet nearly half of those upon whom I have operated for hernia have died, and more than half of those for tracheotomy, and nearly all after trephining. But all those were cases after the operation, not in consequence of it. They were vain, yet quite justifiable, attempts to save lives that, but for the operation, were only yet more certainly lost than they were lost afterwards. These cases, therefore, should not be allowed to swell our tables of mortality. They may be studied most carefully, but they must be studied as special cases where the history of the disease cannot altogether be separated from the history of the operation. And there is yet another class of cases; those, namely, in which patients, after operations, die of diseases that, as it were, fall casually upon them; the deaths, for example, from typhus or typhoid fever, from scarlet fever, from scurvy, and the rest; these should not properly, or without explanation, swell our tables of mortality. Only in dealing with these we need to be very honest.

Sir Astley Cooper used to tell that in going over the wards of a foreign hospital with one of its most distinguished surgeons, the surgeon told him that in his practice amputation at the shoulder-joint, which he had often performed, had never proved fatal. In the course of the same morning, Sir Astley Cooper found in the dead-house the body of a man whose arm had recently been amputated by that surgeon, at the shoulder-joint, with the wound unhealed, and he asked the reason of it. The answer was, "He did not die of amputation, he died of pneumonia," that is, as we should say now, he died of pyæmia, one of the symptoms of which was the pneumonia, which never would have happened to him but for the operation by which his arm was removed. We need, therefore, be very honest in these cases, the more honest because there is so great a temptation to be the reverse, and the fraud is so easy, to assign a death to that which looks as if it were the consequence of something falling by accident upon the patient, although that accident, but for the operation, would never have befallen him. But, setting aside these cases, let us look at those which do really belong to the history of the operation, which it is our chief business to avert; and first of all respecting deaths from shock.—My impression is that they are extremely rare. I can scarcely remember to have seen one that could be fairly assigned only to the shock of an operation, and my supposition is, that these become much more rare now than they were in former times, partly by the influence of anæsthetics, and partly also by the more liberal and more natural way in which we treat our patients before the operation, so that they can come to face the difficulty, not with the least, but with the most strength we can give them.

Nevertheless, such cases do happen, and it would be well if we could so analyse as to be able to assign in each case which particular constituent of a shock was the cause of death; for example, the loss of blood, the great impression of alarm and terror on the mind, the influence of an anæsthetic, the great violence done and impressed, whether consciously or not, on the nervous centres, and from them reflected not form upon the heart alone, but upon every organ of organic life—how does each of these contribute to the general total of a shock? Cases could be adduced where each of them alone has been fatal; but when all concur, as they do in almost every surgical operation, it may be very difficult, and may seem almost impossible, to assign to each its proper share in the general result of great danger or deaths from shock. Yet it is very necessary to study them as if we could decide which has contributed most largely, or which has been alone sufficient, for clearly we have to deal, not with shock as a total result of an operation, but with each shock standing alone. According to its source, so must be the measure of its treatment. My impression is, that in recent times, it has become too customary to regard the loss of blood as the greatest mischief done in producing the shock, and to consider too little the impression upon the nervous system, which being not consciously perceived, through the influence of anæsthetics, is apt to be overlooked.

I would indeed spare blood with all caution; but not so much from the fear of any immediate consequences, as because a large loss of blood seems to make a patient more liable to erysipelas and pyæmia, and the other subsequent mischiefs of an operation, and certainly makes him less capable of recovery from them if they do befall him. But it is still my belief that that which we have most to study in regard to the phenomena of shock, are the results produced by great violence done on the nervous system, of which I observe that they are less considered in the present day, only because we are not aware of them, through not being aware of the amount of suffering endured by the patient under the influence of an anæsthetic. I know no case in which the courage to do little is more necessary than in watching the patient after the shock of an operation; for great energy in treatment may certainly do great mischief. It is quite enough, from whatever source the shock may come, if we can maintain life for a time at a very low level, and if we can do this by the smallest sufficient quantity of stimulants, especially, in these cases, of brandy; for if we can do this, then time and the natural process of events work quietly for the recovery of the patient; and we may be nearly sure that if he can be so maintained, the re-action will, of itself, and almost necessarily, ensue; and we may believe this, notwithstanding a temporary deepening of the influence of shock, for, I think, if others will watch it, as I have done, sitting by and noticing the case almost minute by minute, they will find—and it may be of some interest, even in a physiological inquiry—that the recovery from shock is not a direct and continuous ascent from the deepest depression to the most complete re-action, but rather a vibrating and undulating one.

The patient is now better, and then again, without interference, becomes of himself worse; and as we watch it thus it is rather like the rising of waves in a flood-tide—each one is a little higher than the one that preceded it, until at last there comes the full sweep, and re-action is fully accomplished. And sure I am that a great deal too much interference takes place with shock by those who do thus even minutely watch it, in the fear that the least depression is perilous, and must lead to a yet lower and lower depression still. I have said, too, that the deaths from re-action are probably even less numerous than those from shock. I doubt if I have ever yet seen one of them; and I doubt very much whether genuine re-action, even if it runs up into an acute traumatic fever, needs to be in any way interfered with, or is at all prone to lead into fevers of worse or more perilous type. The larger risks are in those in which re-action is not complete. There are some patients, it will be found, who never rise into a re-action—never rise, that is, after an operation, to anything more than the natural level of health. Now, I am not prepared to say that this is dangerous. I always watch them with great and scrupulous care: for there is, as every surgeon knows, a certain condition in which it may be said that the patient is too well after an operation. He has not recovered even to that discomfort which consists in full re-action.

But many of these will pass by, and may perhaps, for aught I know, illustrate the most perfect method of recovery, where only health is regained. But certainly there is peril when in some patients the re-action is too faltering, faltering more than it should, not only undulating, but each of its waves falling a little more than it rises. Again, there is great peril in re-action that is too long delayed, for certainly the more re-action is delayed the more likely it is to slope into a greater mischief; and certainly these are not cases to be left alone; in all these re-action need be accelerated by stimulants, and brought more nearly to the time at which it should of itself and spontaneously recover. And more perilous still are the re-actions that are attended with hurry and confusion, with disorder, and restlessness, and delirium or convulsions, or with great expenditure of any kind of force—all these are perilous; they are cases of what has been described as action without power. They are difficult to explain physiologically; and yet I suspect most of them would be found to occur in those who have, if I may so speak, a natural instability of constitution; for in all these, as soon as the natural balance and stability of life are disturbed, there seems to ensue a rapid waste of structure, and with that a rapid and purposeless production of force.

For all these there seems to be one great remedy, namely, opium, which acts, I suppose, not merely as an anodyne, but by the singular power which it possesses of hindering waste, and thereby preventing the production of purposeless force, which is certainly the consequence of accelerated waste. Now, the re-actions that I have spoken of have been general. As the shock falls directly or by reflection upon every part of the body, not on the heart alone, observe, but upon every part of the organic life; so, commonly, is the re-action general, and every part has its due of just work in it. But chiefly and especially the force, both of shock and re-action, falls upon the part which is the very seat of injury, the very seat of the operation. Or if great violence be inflicted, there may be for a time total suspension of all nutritive processes, and the part may die; primary gangrene may ensue, or escaping this, after the suspense there may come a much more than normal re-action, a much more than normal exercise of organic force, an acute inflammation; nevertheless, this acute inflammation is never, so far as I have seen in any of the external parts, the consequence of more than local mischief.

We see, for example, after an amputation, or an operation on the face, that great and active inflammation ensues, but it does no other than a local harm. It may spoil the healing of a wound; it may give rise to a recurrent hemorrhage; it may produce too great suppuration; it may even produce a primary local gangrene; but with this it stops, and never, so far as I have seen, leads to death, or to any more than a local calamity. But sometimes, when the operation has been performed upon parts of great extent or of great importance to life, this local re-action, which may be observed completely independent of the general one, may be fatal. Thus, for example, patients die rapidly sometimes after ovariotomy, of a suddenly fast-setting-in peritonitis; and this, although the phenomena of a general re-action may not have been clearly established; and it is to be observed that they die as patients die, with peritonitis after perforation, or as they die sometimes, with rupture of the intestines; they die as if with shock, with a sudden setting-in of extensive and acute disease. Deaths of this kind are growing more rare still than they used to be, for I think we know better now how to treat them. Deaths of the same kind used to occur more often than they do now from lithotomy; patients died then within one or two or three days after the operation, from some sweeping inflammation of the peritoneum or pelvic cellular tissue; and I think they died more commonly then than they do now, when they were treated with bleeding or with other antiphlogistic treatment; for the things to be met are not merely the phenomena of acute inflammation, but the shock which the setting-in of such inflammation produces, or, if I may so speak, re-produces. Before they have recovered from one there comes in another shock, and that shock proves fatal. The treatment for them should be, not by bleeding, but stimulants. Although these cases may have about them even the signs of an acute peritonitis, stimulants and opium must be given until the shock is completely gone by. Now, these cases of which I have spoken—and I might mention some more, especially those of primary and recurrent hemorrhage, as sources of danger or of death—these all might, in the more accurate and minute records to which I wish to move the Society, form a group as it were by themselves, for they all are the results of mischiefs which are, so to speak, the more or less of natural things—of things which we cannot altogether avert; and with these I would class one which comes sometimes in one group and sometimes in another, namely, the use of chloroform. I could not find terms strong enough to speak of the value of anæsthetics for surgical operations; and let me say, not only for their mercy's sake, not only for the saving of pain, but for the abundant good that follows afterwards—the loss of that impression with which patients awake every morning from a disturbed night's sleep, the loss of the reflection and memory of the operation, the great diminution of all that shock that was dependent upon absolute pain, whether the pain were reflected from the nervous system or not.

All these things are so great blessings that one seems to be ashamed to detract in the least measure from the influence of anæsthetics, and yet I must mention one. I have said that great energy of treatment may be a great mischief in the treatment of a shock after an operation. I mean that it is so if after the profuse giving of stimulants or of food of any kind sickness be produced; for among all things which can complicate the depression of a shock vomiting seems to be the worst, and unquestionably this will sometimes follow the influence of anæsthetics, and when it does follow, I know nothing except time by which it can be averted. I know it can be prevented commonly by giving the patient the most light and digestible food, some two or three hours after the administration of the anæsthetic, and by great care over the digestive organs. But notwithstanding these, it will sometimes happen, and it complicates with great severity the occurrences of a shock; for when it begins, so far as I know, medicine has no control whatever over what we call chloroform sickness. I know nothing that will stop it.

It will stop in time; after a few hours in some; after a few days in others. It is of no mischief to all the cases of minor shock; but for all the larger ones it is, unquestionably, a great addition to the peril, and I cannot doubt that it has sometimes been a fatal one. It deserves, therefore, the most careful study of all the members of the Association, and of all that can contribute to its remedy. But when I say this, let me say also that this is the only mischief that I know of it. There are never wanting those who will ascribe to anæsthetics certain deaths which ensue after operations in which the shock inflicted has been comparatively small. Patients have submitted to minor operations, and in a few hours they have been in great peril, and in a day they have died; and it seems to be forgotten entirely that cases of this kind not only occurred, but were, as I believe, much more frequent, before the introduction of anæsthetics. There is an admirable work, which is familiar, I am sure, to all the older members of the Association—Mr. Travers on "Constitutional Irritation;" and there they will find abundant instances of this—where patients, after comparatively the most trivial operations, have died, as we must believe, under the influences of shock; the only singularity of their cases was that the cause was apparently so trivial: but if those cases be referred to, it will be evident that the influence of anæsthetics is excellent in preventing deaths such as these by diminishing what in all probability was the chief cause of shock here—namely, the great mental alarm, pain, and dread of the consequences of the operation.

Now let me pass to another set of cases, which come in larger number, as the results of operation, and which are different from all that I have spoken of hitherto, in that they are not strictly to be called inevitable; they are not, in any sense, the more or less of the necessary consequences of the operation; rather, they come from those things which, it seems to us, if we can be bold enough to cast our eyes forward, it is not only our plain duty, but within our ability to avert—those, namely, I mean, which partake more of the characters of diseases, and which we may believe are severally due to specific morbid alterations in the blood, whether due to mischiefs coming to the patients from without, or to those that are bred within him by the natural results of the operation. It seems to me very important that we should be quite clear in recognising all these cases of erysipelas, of pyæmia, of phlebitis, or nearly all of them, of secondary gangrene, of tetanus, and the rest, as really, and from their beginning, constitutional; as general that is, before they are local; for if we might do so, then we might study them by the light of those which are the very types of these diseases, these acute morbid affections of the blood, namely, eruptive fevers, and study them by the light not only of their pathology, but by that which we have already learned to do in their management or their treatment. It is quite true that erysipelas or phlebitis is most apt to appear at the seat of the operation, and to look, therefore, like a disease of purely local origin, but the same will happen with the truest eruptive fevers.

Some few years ago I cut a boy for the stone; three days afterwards he seemed in great peril of his life, with general disturbance of his system; the day after there appeared a brilliant red eruption at the wound. That was measles—earliest, most intense, at the seat of injury, and thence it spread over the whole body, and passed by without doing damage. I have known the same thing to occur with scarlet fever after an injury at the knee-joint. Dr. William Budd has recorded a similar case of small-pox, after a bruise upon the nates. So that really this local occurrence of erysipelas or other diseases at the seat of operation, is no more proof of their true local origin than in these cases. Measles was not proved to have a local origin because it occurred first and most intensely at the wound of lithotomy. And there is another character, one which seems to me very important in relation to the diagnosis between these diseases that are of true traumatic origin, and purely local, and those which, although they occur at the seat of injury, are yet truly specific, and of blood origin—namely, the difference of time at which the local inflammation will, in the several cases, set in.

A true local and traumatic inflammation comes in either before or very little after the time of general re-action; so that, within one or two days, usually, we find it at the wound, or it may be later—three or four, or in some cases, even five days, when the general re-action has been long delayed. The later it is, the worse. On the same side, that which is a specific blood-disease, comes in after the re-action, and commonly even with considerable interval of time between the two. Thus, for example, we may find not unfrequently after an operation on the face, where these phenomena are best shown, or after an amputation, the next day, or two days later, the parts around the wound may be swollen, œdematous, painful and ruddy, the seats of active inflammation. But this is not erysipelas, nor a disease of any great moment, nor one that will tend to more than local troubles. I have never seen a case of that kind which required any active treatment. But after it is passed, if it has ever occurred, and it may be even many days later, there may set in another inflammation, looking—it may be very like it—with similar swelling, redness, tension and pain, but the later inflammation is sure to be one of erysipelas or pyæmial origin, or in some other form specific. So, too, with phlebitis. On the day after the amputation the femoral vein, for example, may be sharply inflamed, tender and painful, and with some constitutional disturbance; but this is never a disease of great moment; it passes by without treatment. But in that respect it is very different from the phlebitis which may ensue a number of days later, and which is quite surely pyæmial, or connected with some grave affection of the blood. I know, therefore, in these respects, nothing more important than studying the very time at which these inflammations set in. If they be early and only traumatic, they pass by without damage; if they be late, whatever the result, they must be regarded minutely as cases of general pyæmial or erysipelatous origin, indicating something wrong about the patient or within him—something to be amended.

There is yet another character—namely, that inflammations, of however acute a kind, which are traumatic, are very seldom preceded by any appropriate constitutional disturbance. It is remarkable that it should not be, but we never see them thus preceded. On the other side, those that are of general or blood-origin, very rarely ensue without rigors, or some profound affection of the nervous system. In speaking of rigors, if I may diverge so far, I wish I could provoke some one to the more minute study of them, to see what their true physiology is. What is the meaning of that strange shuddering which we see as the precursor of some of the most formidable diseases that we have to deal with? so strange as it is, too, in its relation to the urinary organs, so often the precursor of great mischiefs; so strange in its relation to an accumulation of pus that cannot be discharged. I fear that we are as yet almost wholly ignorant of its physiology; but I would suggest—and let it be my contribution to the study I should like to invite some one to—that we are too much in the habit of thinking that its most important indication is in the sensation of cold which most patients endure with it. Yet this is really only a sensation, and, moreover, only a subjective one; for it is certain that, even before the rigor ensues, the temperature of the surface of the body is increasing, and that it continues to increase during the whole course of the rigor. I would suggest that they should be studied rather in relation to convulsive diseases, and my reason is, that they, not only in a thorough rigor, have all the essential characters of convulsion, but may also, as surgery sometimes unhappily shows, be replaced by convulsions.

Three years ago I operated on a gentleman for stone. Two or three days after the operation he had a terrible rigor, and that was followed by great heat, producing sweatings, and then by extensive suppuration over the surface of the chest. A few days later, another rigor ensued, with similar phenomena subsequent to it, and with other characters of pyæmia, and with a second great suppuration. Some days later than that, he had a severe epileptic seizure, and that was followed in the same way with a profuse suppuration again, in another part of the walls of his chest, and then with various signs of phlebitis, he gradually recovered.

Not long ago a woman was under my care in St. Bartholomew's Hospital, with relapsing erysipelas. All the previous attacks of erysipelas were preceded with definite rigors. A rigor regularly told of the coming relapse of erysipelas, but the last attack was preceded by severe convulsions, and they were followed by three days' coma, and the coma was not relieved until the erysipelas appeared. For the rest of her life she had no further cerebral symptoms, and after her death we found no indication of disease in the brain. She had died only exhausted.

This case has been told me. A member of our profession had chronic pyæmia; in all the earlier part of his illness each suppuration characteristic of it was preceded by a rigor; in the latter part tetanic convulsions preceded each suppuration. I could mention, I think, other instances in which convulsions do thus replace and substitute the ordinary phenomena of a rigor. I imagine, indeed, that those that are familiar to many members of the Association, convulsions that precede eruptive fevers in children, are of the same kind. But this must suffice for the suggestion I have made, and I am diverging too far from the subject that I began with, which was, namely, to indicate by those precursory constitutional symptoms the necessity of distinguishing clearly between those inflammations that are truly local and those that are pyæmial, or of erysipelatous origin, and by that to tell what seems to be a greater need, that we should study all these diseases by the light of the true eruptive fevers; I mean, namely, all cases of erysipelas, of pyæmia, of secondary gangrene, of secondary phlebitis, and even, I would add, of tetanus, and all others that we have to class together as the great source of our mortalities after operation.

But the mere enumeration of these cases must be sufficient to tell that I cannot speak of them all now; only let me take the liberty of mentioning one or two things that have been most upon my mind, as most worthy to be told those that have a large and widely-extended practice, and who might, with their contributions, bring in such a mass of evidence upon the point as has never yet been accumulated. First of all, in regard to the causes of these things. We know very well that among the general external causes for all these mischiefs, there are the crowding of patients into too limited a space, that is, into spaces where they have too limited a supply of air, uncleanliness—in a word, if I might sum it up quickly—dirt of all kinds, within or without; and for internal conditions, we know very well that those patients are most prone to them who are, as we may believe, most unstable in their composition—namely, those whose tissues are disordered, either by defective food, or by intemperance, or by excess of animal food, or those who retain in their frames too large a quantity of refuse—matter not sufficiently excreted—as the gouty, and above all, those that suffer with granular degeneration of the kidneys. We know well enough that in any one of these the ordinary consequences of a surgical operation are sufficient to bring in that morbid condition of the blood which manifests itself in erysipelas, pyæmia, gangrene, or some other source of fatality. But my impression is, that there are yet some other causes to be sought for; for where even we may believe that all these are absent, yet these plagues come in. For example, I do not know that we can assign any one of these diseases to be a product solely of hospital practice. I must say for myself, that I have never seen one of them more intense or more fatal than I had seen it in single cases amongst the best and most well-ordered houses of the metropolis; certainly not one that I could mention which has been worse in St. Bartholomew's Hospital, than I have seen it in a house, in a room, and under a charge with which I could find no distinct fault. It may be said that in hospitals we can never altogether exclude the sources of foulness and infection. I am not prepared to say, I am not prepared to doubt, whether they may not exist also even in our best houses; whether our sanitary arrangements anywhere are so perfect as to shut out the sources of even the worst form of zymotic disease.

I can only suspect that there are yet some things hidden. I cannot yet profess myself prepared to believe that there is no one of us living in such conditions of external health, but that after an operation there is that lying at his bedside which will breed some dire calamity to him. But I would suggest here—not only to hospital practitioners, upon whom, most unjustly, as it seems to me, the whole burden of proof of these cases has been hitherto cast, but to those who practise also in private—that in every case where any one of these diseases occur, I would most conscientiously try, and that we should look for its source to the hospital or the private house, or the practice should be brought to a strict trial—a private trial, if you will—but a just and a true trial before our conscience; and if the hospital or the house, or the practice be found faulty, let it be condemned, and be at once amended. Another point I would suggest is, that it is not enough for us to study these things only in their worst forms, in those in which they are deadly, for we can never know the true extent of a mischief if we know none but those whom it kills. If a patient barely escapes with his life, or even in the process after an operation he passes through any peril, even of the least, he may indicate as great a necessity for amendment of the conditions in which he has been placed, as if he had died. This is true of erysipelas; and there is a thing to be considered, that, seeing how vast are the differences between the worst and best cases, it may be quite within our power to reduce the whole of the cases that do occur to the condition of the lowest peril.

But much more, it seems to me, is this the case with pyæmia, or rather with the group of diseases which we have to include under the most inappropriate of all terms, a term not only insufficient, but altogether false; for the one thing there is not in pyæmia is pus in the blood. Look only at the number of cases that are included in this disease, and the number which altogether elude not our observation, but our record. There are, first, cases of acute pyæmia, well marked by rigors, by profuse sweatings, by rapid exhaustion, by articular suppurations, by pneumonia, deposits of pus here or there, and rapidly fatal. These are the cases that swell up our records of mortality; but there are others which ought to be recorded and studied with equal care, namely, the chronic, where the same series of phenomena ensue, occupying only a longer time for their occurrence; and again, there are yet others as truly pyæmial, those which are seldom recorded at all, namely, those in which there are but threatenings of these conditions. Patients that have repeated rigors, slight pains about the joints, and abscesses at some distant interval of time, pass through their course with that which looks scarcely like peril, and yet it may be the very same disease which, overlooked in the hospital, may cause the deaths of all patients that next come under operation; and again, there are yet others, cases that after an operation pass on with one abscess after another. All are familiar with these, but they are not reckoned commonly as pyæmial. The whole history of their occurrence, and the most accurate comparison of them with others of the same acute type, shows their identity; and there are yet others, where patients, after operation, are attacked with inflammation, first in one vein and then in another, then with induration of the subcutaneous cellular tissue, then with a small deposit of pus that is soon discharged and healed up. These again are cases that we need minute record of, that in all hospital practice should be recorded, in regard to the grave question whether these might be avoided, for I venture to say, if we can avoid these more local manifestions of the poison, so surely we can avoid the more principal ones.

I have said that one advantage of studying these cases by the light which we have from those that are the express and typical examples of blood disease of the acute form, is that you have to treat them after the same method as we now deal with the eruptive fevers. I know, indeed, that with regard to these our medical power can scarcely be spoken of as in any strict sense curative—we manage them.

I wish surgeons could manage pyæmia as well as physicians and general practitioners can manage scarlet fever or eruptive fevers that they have to deal with; but still even they do manage more than cure them. And yet let me suggest that there is great hope that we may discover absolute specifics for these things, when we see that some cases or some symptoms may be cured with apparently a true specific power; for example, the recovery of certain patients from erysipelas under the influence of large doses of iron, so definite, (?) that one must surely speak of it as a true specific for the disease in those cases. No one, I think, has been more disappointed than myself in the endeavor to make iron useful in all cases; but no one can be more convinced that there are certain cases in which iron is a distinct and certain cure for erysipelas. Quinine has, in some cases, a power of the same kind. It cures, as Dr. Latham says, outright, and in the strictest sense. And having occasion to mention that honored name, let me congratulate the members of the Association on the rich treasury of knowledge which he lately contributed to the profession—knowledge which shows him so old in experience, so fresh in intellect, so strong, as he has always been, both in the power of thinking and in the art of telling what he thinks.

And there is another remedy of this kind. Quinine, in large doses, will cure the rigors of an acute pyæmia (?) almost as certainly and as swiftly as it will cure ague; and curing these by what seems to be a distinct and specific power, it will sometimes help materially to the final recovery. But I cannot profess that I have seen it do more than thus help; and if we look back to the records of the many trials and many disappointments I have endured, I think there is but one thing I have ever seen have fair remedial power over pyæmia: that is, a profusion of fresh air. The most remarkable recoveries from pyæmia—they are three—that I have seen, were those in which the patients may be said to have lain day and night in the wind. They lay with the wind blowing all round about them through the room. They so lay, that I have said that I wished they could hang our patients in beds outside of the hospital, rather than within the walls. But it is time I should have done; and though out of the abundance of my ignorance I could go on for a length of time, telling of things that I think require our most careful study, let me only end by impressing on you some of the great motives that we have to make this the chief subject of our study: I mean the means by which we may reduce the mortality after our surgical operations.

I need not dwell upon our selfish interest in the matter; I need not speak of the disappointments and deep regrets that we should save ourselves, if we could only, by some measure, reduce the mortalities of these things; but rather, see that the whole course of surgery of late years has been such as to render the performance of surgical operations more and more feasible, if only we could greatly reduce their mortality. They can be done without pain. The after treatment may be now conducted with so little pain, that I should count it as a matter of mismanagement if, in any ordinary cases, a patient complained at all after the first or second day.

The rules for operation are every year improving; the education of students in them is every year advancing; constantly our mechanical appliances are improving, and drawing more near to perfection; and witnessing the cases of ovariotomy and re-sections of joints and others, we may safely say every year adds to the number of those things that should be done to save life. Yet over all this, which might be so bright, there hangs a cloud—as it were, a dark pall.

We cannot do these things without risk, and often without too great risk to life. Our risks in amputation are counted by the mortalities of from fifteen to fifty per cent.; after amputations of the breast from five to ten per cent.; after lithotomy in the adult, twenty, forty, or fifty per cent.; and even after minor operations the mortality ranges in one's practice, I suspect, to two, or three, or sometimes even more than that per cent.; and it is because of these things that we are driven sometimes to painful substitutes—to caustics, to the ecraseur, and lithotrity; and we learn from abroad of tolerated barbarisms, of practices which might lead us to think that the whole art of dexterous surgery was lost. We learn of limbs amputated and eyes extracted by caustics—of limbs wrenched off, after crushing of bones and crushing through their flesh; and this is justified in the minds of the surgeons—for they are surgeons by whom these things are done—on the plea that a cutting operation is of so great risk that there is no substitute too bad for it.

To amend this is the task that we have before us; and with what heart may we go to the work? Why, surely, with very good heart, if we see what is done in some places, and under the best care, and contrast the results of the best circumstances with those of the worst. Let me cite only three cases. A very distinguished member of our Association, Mr. Hussey, records from Radcliffe Infirmary, Oxford, that the true mortality after amputation is only thirteen per cent., and the mortality in the Parisian hospitals is upwards of fifty per cent.; and he states that of fifty cases of amputation performed in that same infirmary, below the knee, there was but one death. Mr. James records from the Exeter Hospital that the total mortality of all amputations is only fourteen per cent., and amputations after disease have a mortality of less than nine per cent. So Dr. Humphrey again records from a hospital in Cambridge, that the total mortality of amputations, of the lower extremities, observe, is only sixteen per cent.—a mortality which rises in the Parisian hospitals, which are far from being the worst in Europe, to upwards of sixty per cent.

These are the things which have been achieved, and these are the things which it is the express duty of every hospital and private surgeon to see that he achieves in his own practice; for we should go to the study of these things with the consciousness that a great part of the deaths that still remain are not inevitable; that by more care and scrupulous attention, not perhaps in our own practice on the very patient himself, but in all that surrounds him, these mortalities might be reduced. Even in Paris, within the last twenty years, the mortalities from great operations have been reduced by at least ten per cent.

Now as much, and much more than that, must be done in England; and so much, and much more than that, will be done, if the members of the Association will say that it shall be done, and will act vigorously on the decision.

